# Efficient Constitutive Expression of Cellulolytic Enzymes in *Penicillium oxalicum* for Improved Efficiency of Lignocellulose Degradation

**DOI:** 10.4014/jmb.2101.01003

**Published:** 2021-03-26

**Authors:** Pankajkumar Ramdas Waghmare, Pratima Pankajkumar Waghmare, Liwei Gao, Wan Sun, Yuqi Qin, Guodong Liu, Yinbo Qu

**Affiliations:** 1 State Key Laboratory of Microbial Technology, Shandong University, Shandong 266237, P. R. China; 2 Tobacco Research Institute of Chinese Academy of Agricultural Sciences, Shandong 266101, P. R. China; 3 National Glycoengineering Research Center, Shandong University, Shandong 266237, P. R. China

**Keywords:** *Penicillium oxalicum*, lignocellulose, constitutive expression system, cellulase, enzymatic conversion

## Abstract

Efficient cellulolytic enzyme production is important for the development of lignocellulosedegrading enzyme mixtures. However, purification of cellulases from their native hosts is time- and labor-consuming. In this study, a constitutive expression system was developed in *Penicillium oxalicum* for the secreted production of proteins. Using a constitutive polyubiquitin gene promoter and cultivating with glucose as the sole carbon source, nine cellulolytic enzymes of different origins with relatively high purity were produced within 48 h. When supplemented to a commercial cellulase preparation, cellobiohydrolase I from *P. funiculosum* and cellobiohydrolase II from *Talaromyces verruculosus* showed remarkable enhancing effects on the hydrolysis of steam-exploded corn stover. Additionally, a synergistic effect was observed for these two cellobiohydrolases during the hydrolysis. Taken together, the constitutive expression system provides a convenient tool for the production of cellulolytic enzymes, which is expected to be useful in the development of highly efficient lignocellulose-degrading enzyme mixtures.

## Introduction

Lignocellulosic biomass is an abundant and renewable organic resource [1]. Bioconversion of lignocellulosic biomass to biofuels and chemicals is helpful to diminish the dependency on depleting petroleum reserves [2]. However, the commercial bioconversion of lignocellulose to biofuels is still challenging due to the heterogeneous and recalcitrant nature of lignocellulosic materials [3, 4]. Thus, a pretreatment step prior to enzymatic hydrolysis is required to alter the structure and composition of lignocellulose and, therefore, improve the efficiency of hydrolysis [5], and efficient lignocellulolytic enzyme mixtures need to be developed to lower the cost of the process [6].

Cellulose is an unbranched polysaccharide comprised of glucose subunits linked by β-1,4-glycosidic bonds and is the major constituent of most plant cell walls [7]. Generally, the enzymatic hydrolysis of cellulose involves the synergistic action of three types of core hydrolytic enzymes, namely endo-β-1,4-glucanase (EG, EC number 3.2.1.4), cellobiohydrolase (CBH, 3.2.1.176/3.2.1.91) and β-glucosidase (BGL, 3.2.1.21), collectively known as cellulases [3]. Currently, filamentous fungi are mainly used for the industrial production of cellulases. For fungal cellulases, the EGs mostly act on internal bonds in the cellulose fiber and generates free chain ends. CBHs are processive cellulases hydrolyzing cellulose chain to cellobiose by acting on reducing ends (CBHI) or non-reducing ends (CBHII), while BGLs hydrolyze cellodextrins into glucose [8, 9]. Additionally, lytic polysaccharide monooxygenases (LPMOs, 1.14.99.54/1.14.99.56) break down cellulose using an oxidative mechanism, which can significantly enhance the action of hydrolytic enzymes [10].

Supplementation of exogenous enzymes and *de novo* design of enzyme cocktails are effective approaches to improve the performance of cellulase mixtures [6, 11]. Previous studies on various cellulolytic fungi have reported a set of highly active cellulases capable of degrading crystalline cellulose more efficiently than the components in common commercial cellulases [12-15]. Many of the enzymes used in these studies were purified from the culture supernatant of their native hosts, which is a time-consuming and labor-intensive process. Therefore, the efficient production of proteins in well-established host strains is valuable for the investigation of cellulase components. Particularly, filamentous fungi are more suitable for the expression of some cellulases than bacteria and yeasts regarding their properties [16, 17], which highlights the importance of developing expression systems using natural cellulolytic fungal species.

*Penicillium* strains have been reported for the production of balanced lignocellulose-degrading enzyme systems with superior performances. Therefore, they are considered as potential alternatives to the industrial cellulase-producer *Trichoderma reesei* [18, 19]. *Penicillium oxalicum* strains are commonly isolated from the natural environment for their high cellulase-producing capacities [20, 21]. Genomic and proteomic analyses of a *P. oxalicum* wild-type strain 114-2 have revealed that it produces a lignocellulolytic enzyme system rich in hemicellulases [22]. Some *P. oxalicum* mutant strains have been used in industrial applications for cellulase production and lignocellulosic biomass hydrolysis [23]. Nevertheless, the cellulase system of *P. oxalicum* still needs to be improved in regard to the efficiency of crystalline cellulose degradation [24].

In this study, a constitutive protein expression system enabling the production of relatively pure cellulases was developed using *P. oxalicum*. Nine cellulolytic enzymes of different origins were expressed in this system and then investigated for their abilities to boost the efficiency of *P. oxalicum* cellulases in lignocellulose degradation.

## Materials and Methods

### Lignocellulosic Biomass and Enzymes

Sweet sorghum vinasse produced from continuous solid-state fermentation technology [25] was provided by Hongli Biotechnology Co. Ltd. (China). The vinasse was pretreated with 15% (w/v) ammonium sulfite with a solid to liquid ratio of 1:4 (w/v) for 1 h at 170°C. After the pretreatment, the solid biomass was collected and washed 8 times with distilled water to remove free sugar. A steam-exploded corn stover (SECS) was provided by COFCO Bio-chemical and Bio-energy Co. Ltd. (China). Commercial cellulase preparation SP in the form of solid powder was provided by Sino Biotechnology Co., Ltd. (China) [26].

### Construction of Plasmids and Strains

To construct the gene expression vector pP*ubiD*-*pyrG*, the selectable marker gene *pyrG* (orotidine-5'-phosphate decarboxylase) from *Aspergillus nidulans* was inserted into the *Kpn*I/*Eco*RI site of plasmid pUC19 (Tsingke, China) to generate plasmid pUC19-*pyrG*. The *P. oxalicum* *ubiD* gene promoter and *A. nidulans* *trpC* gene terminator were then fused together, with a *Bam*HI site introduced between them. The fusion product was inserted and removed the *Bam*HI site of pUC19-*pyrG* to generate plasmid pP*ubiD*-*pyrG*. The cellulase genes from *T. reesei* and *P. oxalicum* were amplified from the genomic DNA of corresponding strains QM9414 and 114-2, respectively. The other cellulase genes were synthesized by Genewiz (China). The cellulase genes were inserted to the *Bam*HI site of pP*ubiD*-*pyrG* to produce gene-specific expression vectors. The cloning operations were performed using the ClonExpress II One Step Cloning Kit (Vazyme Biotech, China) according to the manufacturer’s protocol. *Escherichia coli* DH5α (Tsingke, China) was used for plasmid transformation and amplification. All the primers and their uses are listed in Table S1. The nucleotide sequences of cellulase genes are shown in Table S2.

The cellulase gene expression vectors were linearized using *Nde*I and transformed into *P. oxalicum* uracil auxotrophic strain M12 (*pyrG*^-^) [27]. PEG-mediated protoplast transformation was performed as previously described [28]. The purified transformants were confirmed by diagnostic PCR and sequencing.

### Enzyme Production

The correct fungal transformants were grown on wheat bran liquor slants at 30°C for 4 d, and the spores were then collected in 0.9% (w/v) NaCl solution supplemented with 0.05% (w/v) Tween 80. The fungal spore suspension was inoculated to seed medium (Vogel’s salt solution [28] supplemented with 20 g/l glucose and 1 g/l peptone) and incubated at 30°C with rotary shaking at 200 rpm for 24 h. The pre-culture was inoculated into 50 ml fermentation medium (Vogel’s salt solution supplemented with 20 g/l glucose) with an inoculation ratio of 10% (v/v), and cultivated under the same conditions for 48 h. For the production of TtAA9E, CuSO_4_·5H_2_O at a final concentration of 0.5 mg/l was added to fermentation media. Uracil at a final concentration of 0.5 g/l was added to the medium if necessary. The culture was vacuum filtered through a 0.45 μm polyethersulfone membrane (Xingya, China). As indicated in the text, the filtrates were concentrated by ten folds using a Macrosep Advance Centrifugal Device 30K (10K for TtAA9E; USA).

### Enzyme Assay, Protein Concentration Determination and SDS-PAGE

Unless otherwise specified, endoglucanase activity (CMCase activity) was measured at 50°C using 1% (w/v) sodium carboxymethyl cellulose as previously described [28]. One unit of enzyme activity was defined as the amount of enzyme required to liberate 1 μM of glucose equivalent per minute under the standard assay conditions. For TaEG, the optimum temperature was determined by measuring CMCase activities at different temperatures, and thermostability was studied by incubating the enzyme at different temperatures for 1 h and then measuring the CMCase activity at 50°C. Protein concentration was determined by using a Bradford reagent kit (Sangon, China). Sodium dodecyl sulfate-polyacrylamide gel electrophoresis (SDS-PAGE) was performed using 12.5% (w/v) polyacrylamide gels, and proteins were stained with Coomassie Brilliant Blue R-250 (Sangon).

### Liquefaction of Biomass

The reaction system in 100 ml flasks contained sodium acetate buffer (50 mM, pH 4.8) and pretreated sweet sorghum vinasse at a loading of 10% (w/w) dry matter (DM), with a total weight of 20 g. TaEG was added at a dosage of 2 mg/g DM. The liquefaction system was incubated at stated temperatures with a shaking speed of 200 rpm.

### Enzymatic Hydrolysis

The hydrolysis system with a total weight of 30 g contained sodium acetate buffer (50 mM, pH 4.8), 0.1% (w/w) sodium benzoate, and 5% (w/w) DM SECS. For the cellulase supplementation experiment, cellulase preparation SP of 10 mg/g DM and a single cellulase component of 2 mg/g DM were added to the system. For the cellulase mixture experiment, simplex lattice mixture design was performed using the Design-Expert 8.0 software (Stat-Ease Inc., USA), with the sum of the three enzyme components always being 10 mg/g DM. The hydrolysis was performed in 100 ml flasks incubated at 50°C with a shaking speed of 200 rpm. The hydrolysate samples were centrifuged at 2348 g for 10 min, and the concentration of glucose in the supernatant was measured using an SBA-40C biosensor analyzer (BISAS, China).

## Results and Discussion

### Construction of the Constitutive Expression Vector pP*ubiD*-*pyrG*

**Fig. 1:**
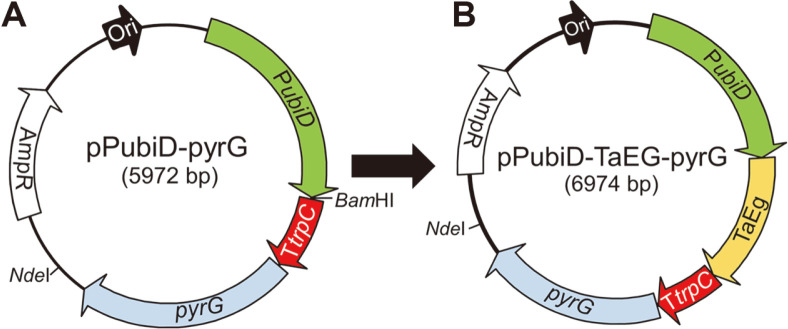
Construction of gene expression vectors. (**A**) The map of empty vector pP*ubiD*-*pyrG*. The *Bam*HI site was used for the insertion of target genes. (**B**) The map of TaEG expression vector pP*ubiD*-TaEG-*pyrG*. The *Nde*I site was used for plasmid linearization.

Similar to many other cellulolytic fungi, the expression of endogenous lignocellulolytic enzymes in *P. oxalicum* is repressed by glucose [22, 29], which provides a low background for extracellular protein production. Therefore, a constitutive gene expression vector was designed for the expression of secreted proteins in the glucose medium. The vector pP*ubiD*-*pyrG* contains a constitutive strong promoter P*ubiD* from a polyubiquitin gene [30]. A *Bam*HI site was added downstream of P*ubiD*, which allows the insertion of the target gene using recombinational cloning methods (Fig. 1A).

### Expression and characterization of recombinant TaEG

**Fig. 2:**
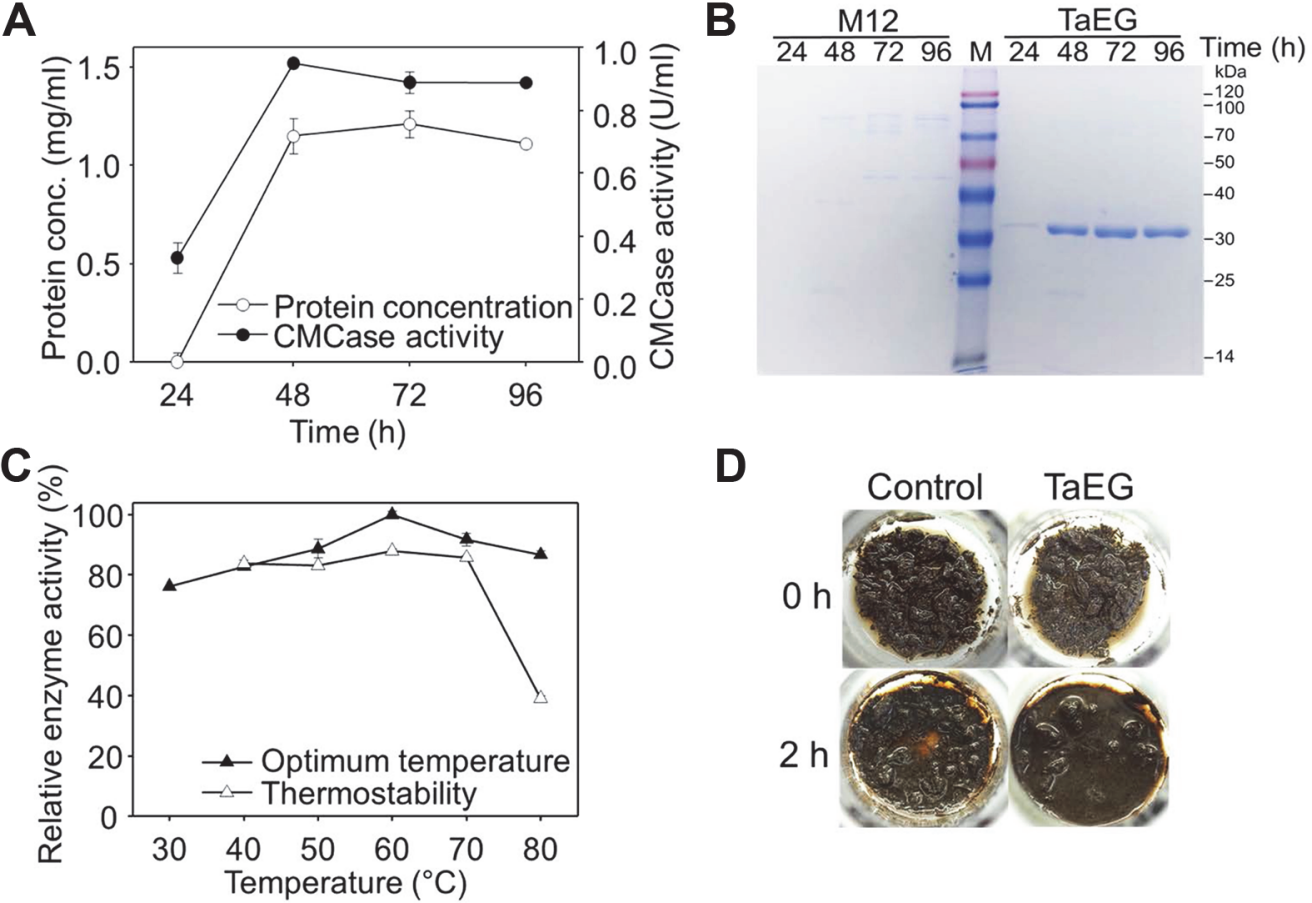
Expression and characterization of recombinant TaEG in *P. oxalicum*. (**A**) Protein concentration and CMCase activity in the culture supernatant of TaEG-expressing strain. Data represent mean±SD from duplicate cultivations. (**B**) SDS-PAGE analysis of the culture supernatants of TaEG-expressing strain and parent strain M12. (**C**) Optimum temperature and thermostability of recombinant TaEG. Data represent mean±SD from duplicate reactions. (**D**) Liquefaction of ammonium sulfite-pretreated sweet sorghum vinasse by recombinant TaEG of 2 mg/g DM at 60°C.

As a first attempt, the gene encoding TaEG from *Thermoascus aurantiacus* (Table 1) was inserted to the *Bam*HI site on plasmid pP*ubiD*-*pyrG* to generate pP*ubiD*-TaEG-*pyrG* (Fig. 1B). The latter plasmid was linearized and transformed into uracil auxotrophic strain *P. oxalicum* M12. The gene expression cassette was expected to be integrated into the chromosomal DNA via random insertion. The correct transformants were cultivated in the medium with glucose as the sole carbon source, and the one producing the highest level of TaEG was selected for further studies. After 48 h of cultivation, CMCase activity of 0.95 U/ml and protein concentration of 1.15 mg/ml were detected in the culture supernatant, which were much higher than those at 24 h (Fig. 2A). SDS-PAGE showed that a single protein band was present in the culture supernatant of the TaEG-expressing strain (Fig. 2B). The apparent molecular weight was in agreement with the predicted value of the mature protein (33.71 kDa). Interestingly, several protein bands were detected in the culture supernatant of the parent strain M12 but not in the TaEG-expressing strain, which is possibly because of the competition between TaEG and background proteins on cellular resources.

The optimum temperature for the CMCase activity of crude recombinant TaEG was 60°C, with a relative activity of 92% detected at 70°C (Fig. 2B). Approximately 80% of activity was retained after incubation at 70°C for 2 h, which is consistent with the TaEG properties reported by Hong *et al*. [31]. When the recombinant TaEG was added to ammonium sulfite-pretreated sweet sorghum vinasse, the substrate was efficiently liquefied after 2 h of incubation at 60°C (Fig. 2D), which is helpful for the saccharification at high solids loadings [32]. Taken together, recombinant TaEG with relatively high purity and reported thermostability was successfully produced using the constitutive expression system.

### Expression of Cellulolytic Enzymes of Different Origins

**Fig. 3:**
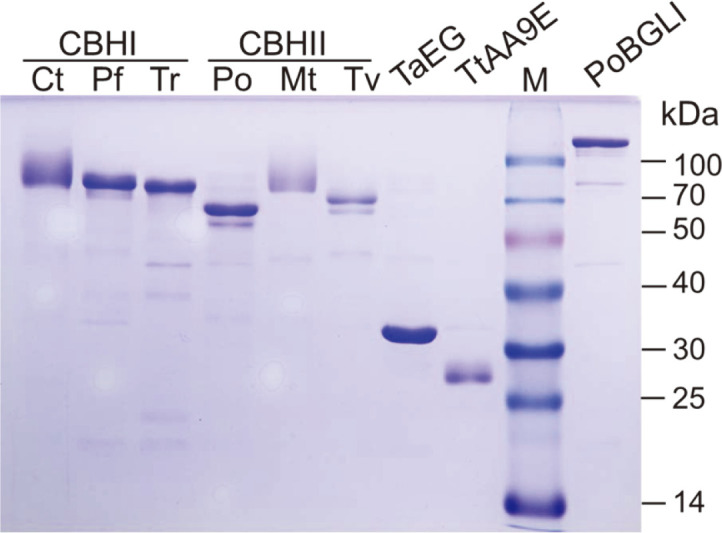
SDS-PAGE analysis of recombinant cellulases. The culture supernatants were concentrated by ultra-filtration and loaded into the gel. M, molecular weight marker.

Considering the effective production of TaEG, cellulolytic enzymes of different types and origins were expressed using the same method, respectively. These enzymes include three CBHI/Cel7A proteins, three CBHII/Cel6A proteins, one LPMO (TtAA9E), and one BGL (Table 1). In the glucose medium, all the enzymes were expressed and secreted into the culture (Fig. 3). The addition of extra CuSO_4_ was found to be essential for efficient production of TtAA9E, suggesting that the copper ion is required for its correct folding or stabilization [33, 34]. Although background proteins were detected in some samples, the purity of these proteins was satisfactory for their application in lignocellulose degradation. All the enzymes, except for TaEG, exhibited apparent molecular weights larger than the predicted values (Table 1), which was attributed to the glycosylation of proteins. *N*-linked and *O*-linked glycosylation types have been frequently reported in the context of fungal cellulases [35]. For example, glycans on the catalytic domain and linker peptide of *T. reesei* CBHI led to 9 to 17 kDa increases in the molecular weight [36]. For CBHI from *Chaetomium thermophilum* and CBHII from *Myceliophthora thermophila*, broad protein bands were detected by SDS-PAGE, suggesting the heterogeneity of their glycosylation [37].

### The Effect of Enzyme Supplementation on the Hydrolytic Efficiency of *P. oxalicum* Enzymes

The heterologous cellulases selected for expression in this study have been reported to have the advantages of high catalytic efficiency and/or high thermostability (Table 1). For example, PfCBHI from *P. funiculosum* hydrolyzes cellulose faster than TrCBHI from *T. reesei* [13]. Therefore, the recombinant proteins were supplemented to the commercial cellulase preparation SP to examine whether the hydrolytic efficiency on the SECS could be improved. The recombinant proteins were loaded at 2 mg/g DM, which was 20% of that of SP (10 mg/g DM). As a control, SP of 12 mg/g DM was also used for hydrolysis, which allowed the comparison of hydrolytic efficiencies at the same protein dosage.

**Fig. 4:**
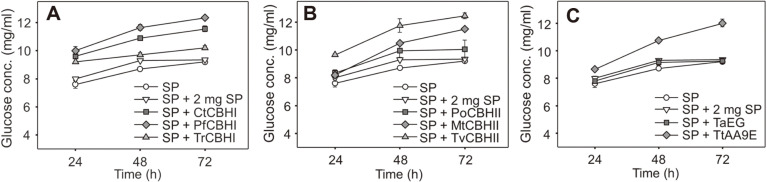
Hydrolysis of SECS using commercial cellulase preparation SP supplemented with different cellulase components. SP enzyme of 10 mg/g DM supplemented with a single cellulase component of 2 mg/g DM were used for the hydrolysis. SP enzyme of 10 mg/g DM without supplementation, or supplemented with SP of 2 mg/g DM, were used as controls. Data represent mean±SD from duplicate reactions. (**A**) CBHI supplementation. (**B**) CBHII supplementation. (**C**) TaEG or TtAA9E supplementation.

As shown in Fig. 4A, the supplementation of all three CBHI proteins were able to enhance the hydrolysis compared to SP enzyme of the same dosage. Among them, PfCBHI was the most effective, with a glucose concentration of 12.35 g/l detected in the hydrolysate at 72 h, 32% higher than the control (Fig. 4A). The superior activity of PfCBHI towards crystalline cellulose over TrCBHI has been mainly attributed to two important motifs in the catalytic domain, as per the results of mutation experiments [13]. Of note, sequence alignment revealed that CtCBHI is more similar to TrCBHI at these two positions (data not shown), which might explain its lower efficiency, compared to that of PfCBHI. The three CBHII proteins and TtAA9E also showed significant enhancing effects on the hydrolysis (Figs. 4B and 4C). Particularly, TvCBHII from *Talaromyces verruculosus* (*P. verruculosum*) showed the highest efficiency, which improved the hydrolysis to a similar extent to that of PfCBHI. However, the structural basis of the high activity of TvCBHII remains to be resolved through domain-swapping and sequence mutation experiments. In contrast to CBHs and TtAA9E, no enhancing effect was observed when TaEG was supplemented to the hydrolysis system (Fig. 4C).

Increasing the dosage of SP from 10 to 12 mg/g DM resulted in faster hydrolysis but not higher glucose yield at 72 h (Fig. 4), implying that the enzyme preparation has difficulty in degrading a highly recalcitrant cellulose fraction in the SECS [24]. The results of enzyme supplementation suggested that the SP enzyme lacks adequate CBH and LPMO activities for the deep hydrolysis of the substrate, while the EG activity was sufficient under the experimental condition. Additionally, supplementation of PfCBHI and TvCBHII remarkably improved the release of glucose in the first 24 h, which was consistent with their high catalytic efficiencies [15]. In summary, the study has identified effective cellulase components for enhancing the hydrolytic efficiency of SP. In the future, genes encoding these enzymes could be introduced into the high-producing strains of *P. oxalicum* for the production of highly efficient lignocellulolytic enzyme mixtures.

### Synergistic Effect of PfCBHI and TvCBHII in the Hydrolysis of Lignocellulosic Biomass

**Fig. 5:**
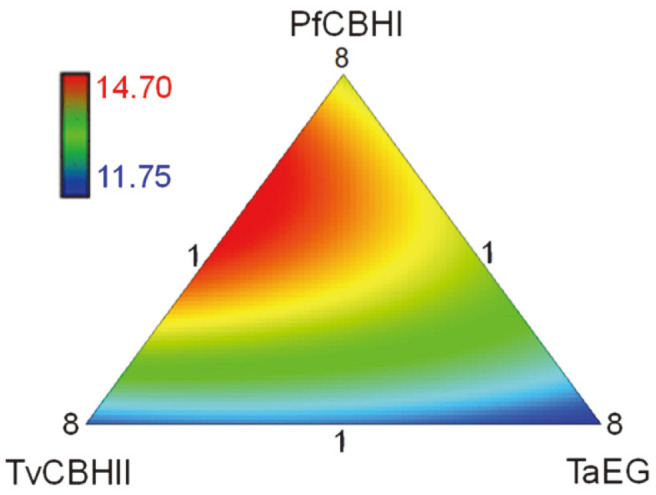
Ternary contour plot of the glucose yields produced by cellulase mixtures. SECS with 5% DM was hydrolyzed for 72 h.

The above results revealed that both PfCBHI and TvCBHII could significantly improve the hydrolytic efficiency of commercial enzyme SP. To provide further guidance for the development of lignocellulolytic enzyme mixtures, the recombinant enzymes PfCBHI, TvCBHII and TaEG were mixed together and used for the hydrolysis of SECS. The artificial enzyme mixtures of different compositions were loaded at 10 mg/g DM (Table 2). To avoid the inhibition of cellulase by cellobiose, PoBGLI of 2 mg/g DM was added to all the hydrolysis systems. Generally, the enzyme mixtures showed similar or higher hydrolytic efficiencies compared to those of SP supplemented with single cellulase components (Fig. 4). Among the ten kinds of enzyme mixtures, number 7 and 8 produced the highest yields of glucose, while the yields of number 3 and 4 were the lowest. Therefore, PfCBHI and TvCBHII played important roles in the hydrolysis of SECS. Moreover, the two CBH proteins had a synergistic effect on the degradation (Fig. 5). The synergism between CBHI and CBHII with regard to crystalline cellulose has been reported since 1980 [38]; however, the mechanism underlying this phenomenon has remained unclear. A recent study by Badino *et al*. suggested that this exo-exo synergy is likely due to their different substrate specificities involving the cellulose-binding domain and linker, rather than their weak endo-acting activities [39].

A synergistic effect was hardly observed between CBH proteins and TaEG, probably because the dosage of TaEG in all combinations (> 1 mg/g DM) was sufficient for the hydrolysis. Nevertheless, the TaEG used in this study belongs to glycosyl hydrolase family 5 [40], while an EG belonging to family 7 has been reported to be important for the hydrolysis of steam exploded wheat straw [41]. Therefore, family 7 EGs could be included in the study of artificial cellulase mixtures in the future.

In conclusion, in this study, we have developed a constitutive expression system for the fast and convenient production of cellulolytic enzymes. The produced enzymes have relatively high purity, which enabled further characterization of enzymes and the construction of artificial cellulase mixtures. Using this system, two CBH proteins and an LPMO were found to remarkably boost the hydrolytic efficiency of a commercial cellulase preparation; of note, they are expected to be co-expressed in production strains in the future. The results provide direction to genetically engineer strains for on-site production of highly efficient lignocellulolytic enzymes.

## Supplemental Materials



Supplementary data for this paper are available on-line only at http://jmb.or.kr.

## Figures and Tables

**Table 1:** Information of cellulases expressed in this study.

Protein name	Origin	GenBank Accession No.	CAZy family	MW (kDa)^a^	Reference
CtCBHI	*Chaetomium thermophilum*	CAM98448.1	GH7, CBM1	54.56	[12]
PfCBHI	*Penicillium funiculosum*	4XEB_A	GH7, CBM1	52.43	[13]
TrCBHI	*Trichoderma reesei*	EGR44817.1	GH7, CBM1	52.24	[42]
PoCBHII	*Penicillium oxalicum*	EPS32164.1	CBM1, GH6	46.56	[22]
MtCBHII	*Myceliophthora thermophila*	AEO55787.1	CBM1, GH6	49.48	[43]
TvCBHII	*Talaromyces verruculosus*	APE61639.1	CBM1, GH6	45.70	[43]
TaEG	*Thermoascus aurantiacus*	AAL88714.2	GH5	33.71	[31]
TtAA9E	*Thielavia terrestris*	ACE10234.1	AA9	22.55	[44]
PoBGLI	*Penicillium oxalicum*	EPS27792.1	GH3	91.55	[45]

^a^Molecular weight predicted using the mature protein sequences without signal peptide

**Table 2:** Hydrolysis of SECS by different enzyme mixtures. Data represent mean±SD from duplicate reactions.

Mixture number	Enzyme dosage (mg/g DM)			Glucose concentration (g/l)		

	PfCBHI	TvCBHII	TaEG	24 h	48 h	72 h
1	2.17	2.17	5.67	11.50±0.14	11.70±0.42	13.00±0.28
2	1.00	4.50	4.50	10.65±0.21	11.15±0.64	12.10±0.00
3	1.00	1.00	8.00	9.95±0.35	10.70±0.00	11.75±0.49
4	1.00	8.00	1.00	9.90±0.42	10.80±0.42	12.05±0.07
5	2.17	5.67	2.17	11.10±0.28	12.00±0.42	13.65±0.21
6	8.00	1.00	1.00	11.80±0.28	12.55±0.35	13.70±0.14
7	4.50	4.50	1.00	12.45±1.06	12.90±0.42	14.65±0.78
8	5.67	2.17	2.17	11.80±0.71	12.70±0.14	14.70±1.27
9	4.50	1.00	4.50	12.00±0.85	12.00±0.42	13.65±0.07
10	3.33	3.33	3.33	11.45±0.07	12.40±0.14	13.45±0.64
