# Supplementary material for: Efficient Constitutive Expression of Cellulolytic Enzymes in Penicillium oxalicum for Improved Efficiency of Lignocellulose Degradation
Source: J Microbiol Biotechnol. 2021 Mar 26;31(5):740–6. doi: 10.4014/jmb.2101.01003 (PMC9705867; doi:10.4014/jmb.2101.01003)
Supplement: Supplementary file 1 [file jmb-31-5-740-supple.pdf]

J. Microbiol. Biotechnol.
https://doi.org/10.4014/jmb.2101.01003

Table S1. List of primers used in this study.

Primer name	Primer sequence (5' to 3')	Product/Purpose
AnpyrG-F19	CTCTAGAGGATCCCCGATCGAT**GCAACTTCCTCGAGAACGCG**	*A. nidulans pyrG* cassette
AnpyrG-R19	TAAAACGACGGCCAGTGAATTC**CCCTTTTAGTCAATACCGTTAC**	
PubiD-F	TGCAGGTCGACTCTAGA**TCTTCATTCCTCACCGAACTCA**	P*ubiD*
PubiD-R	ACGTTAAGTGGATCGGATCC**GGTGACGGCAGTCGTAAGAATG**	
TtrpC-F	CGACTGCCGTCACCGGATCC**GATCCACTTAACGTTACTGAAA**	T*trpC*
TtrpC-R	GAAGTTGCATCGATCGG**AACCCAGGGCTGGTGACGGAA**	
CtCBHI-Fub	CTTACGACTGCCGTCACC**ATGATGTATAAGAAGTTCGCC**	*Ct cbh1*
CtCBHI-Rub	GTAACGTTAAGTGGATC**TTACAGGCACTGGCTGTACCAG**	
PfCBHI-Fub	CTTACGACTGCCGTCACC**ATGTCTGCCTTGAACTCTTTC**	*Pf cbh1*
PfCBHI-Rub	GTAACGTTAAGTGGATC**TTACAAACATTGAGAGTAGTAAG**	
TrCBHI-Fub	CTTACGACTGCCGTCACC**ATGTATCGGAAGTTGGCCGTC**	*Tr cbh1*
TrCBHI-Rub	GTAACGTTAAGTGGATC**TTACAGGCACTGAGAGTAGTAAG**	
PoCBHII-Fub	CTTACGACTGCCGTCACC**ATGCAGAGAACATCAGCTTGGG**	*Po cbh2*
PoCBHII-Rub	GTAACGTTAAGTGGATC**TTAGAAGCTTGGGTTGGCGTTG**	
MtCBHII-Fub	CTTACGACTGCCGTCACC**ATGGCCAAGAAGCTTTTCATC**	*Mt cbh2*
MtCBHII-Rub	GTAACGTTAAGTGGATC**TTAGAAGGGCGGGTTGGCG**	
TvCBHII-Fub	CTTACGACTGCCGTCACC**ATGCTGCGATATCTTTCCATTG**	*Tv cbh2*
TvCBHII-Rub	GTAACGTTAAGTGGATC**CTAAGCCAAAGCTGGATTGGC**	
TaEG-Fub	CTTACGACTGCCGTCACC**ATGAAGCTCGGTAGCCTCGTC**	*Ta eg*
TaEG-Rub	GTAACGTTAAGTGGATC**CTAGAGATAGGGGGTGAGGATG**	
TtAA9E-Fub	CTTACGACTGCCGTCACC**ATGCTCGCCAACGGCGCCA**	*Tt AA9E*
TtAA9E-Rub	GTAACGTTAAGTGGATC**CTAACAGGAGAAG**	

	**ACGGCGG**	
PoBGL-Fub	CTTACGACTGCCGTCACC**ATGAAGCTCGAGTGGCTGGAA**	*Po bgl1*
PoBGL-Rub	GTAACGTTAAGTGGATC**TTACTGCACCTTGGGCAGATC**	
PubiD-CF	CTTCTCGCTACCCATCTCTTTC	PCR verification of transformants
TtrpC-CR	CAACTCCGGAGCTGACATCG	
P15Aseq-R	TGCTGCTTGGACAAATGAAC	

Table S2. Sequences of cellulase genes expressed in this study.

Protein name	Gene sequence[a]
CtCBHI	ATGATGTATAAGAAGTTCGCCGCTCTCGCCGCCCTCGTGGCTGGCGCCTCCG<br>CCCAGCAGGCTTGCTCCCTCACCGCTGAGAACCACCCTAGCCTCACCTGGAA<br>GCGCTGCACCTCTGGCGGCAGCTGCTCGACCGTGAACGGCGCCGTCACCAT<br>CGATGCCAACTGGCGCTGGACTCACACCGTCTCCGGCTCGACCAACTGCTAC<br>ACCGGCAACCAGTGGGATACCTCCCTCTGCACTGATGGCAAGAGCTGCGCC<br>CAGACCTGCTGCGTCGATGGCGCTGACTACTCTTCGACCTATGGTATCACCA<br>CCAGCGGTGACTCCCTGAACCTCAAGTTCGTCACCAAGCACCAGTACGGCA<br>CCAACGTCGGCTCCCGTGTCTATCTGATGGAGAACGACACCAAGTACCAGA<br>TGTTCGAGCTCCTCGGCAACGAGTTCACCTTCGATGTCGATGTCTCCAACCT<br>GGGCTGCGGTCTCAACGGCGCCCTCTACTTCGTTTCCATGGATGCTGATGGT<br>GGCATGAGCAAATACTCTGGCAACAAGGCTGGCGCCAAGTACGGTACCGGC<br>TACTGCGATGCTCAGTGCCCGCGCGACCTCAAGTTCATCAACGGCGAGGCC<br>AACGTTGGGAACTGGACCCCCTCGACCAACGATGCCAACGCCGGCTTCGGC<br>CGCTATGGCAGCTGCTGCTCTGAGATGGATGTCTGGGAGGCCAACAACATG<br>GCTACTGCCTTCACTCCTCACCCTTGCACCACCGTTGGCCAGAGCCGCTGCG<br>AGGCCGACACCTGCGGTGGCACCTACAGCTCTGACCGCTATGCTGGTGTTTG<br>CGACCCTGATGGCTGCGACTTCAACGCCTACCGCCAAGGCGACAAGACCTT<br>CTACGGCAAGGGCATGACTGTCGACACCAACAAGAAGATGACCGTCGTCAC<br>CCAGTTCCACAAGAACTCGGCTGGCGTCCTCAGCGAGATCAAGCGCTTCTA<br>CGTCCAGGACGGCAAGATCATTGCCAACGCTGAGTCCAAGATCCCCGGCAA<br>CCCCGGAAACTCCATTACCCAGGAGTATTGCGATGCCCAGAAGGTCGCCTT<br>CAGTAACACCGATGACTTCAACCGCAAGGGCGGTATGGCTCAGATGAGCAA<br>GGCCCTCGCAGGCCCCATGGTCCTGGTCATGTCCGTCTGGGATGACCACTAC<br>GCCAACATGCTCTGGCTCGACTCGACCTACCCCATCGACCAGGCCGGCGCC<br>CCCGGCGCCGAGCGCGGTGCTTGCCCGACCACCTCCGGTGTCCCTGCCGAG<br>ATCGAGGCCCAGGTCCCCAACAGCAACGTCATCTTCTCCAACATCCGTTTCG<br>GCCCCATCGGCTCGACCGTCCCTGGCCTTGACGGCAGCAACCCCGGCAACC<br>CGACCACCACCGTCGTTCCTCCCGCTTCTACCTCCACCTCCCGTCCGACCAG<br>CAGCACTAGCTCTCCCGTTTCGACCCCGACTGGCCAGCCCGGCGGCTGCACC<br>ACCCAGAAGTGGGGCCAGTGCGGCGGTATCGGCTACACCGGCTGCACTAAC<br>TGCGTTGCTGGCACCACCTGCACTCAGCTCAACCCCTGGTACAGCCAGTGCC<br>TGTAA
PfCBHI	ATGTCTGCCTTGAACTCTTTCAATATGTACAAGAGCGCCCTCATCTTGGGCT<br>CCTTGCTGGCAACAGCTGGTGCTCAGCAAATTGGTACTTATACTGCTGAAAC<br>CCATCCCTCTCTGAGCTGGTCTACTTGCAAATCGGGTGGTAGCTGCACCACA<br>AACTCCGGTGCCATTACGTTAGATGCCAACTGGCGTTGGGTCCATGGTGTCA<br>ATACCAGCACCAACTGCTACACTGGCAACACTTGGAATAGCGCCATCTGCG<br>ACACTGATGCATCCTGTGCCCAGGACTGTGCTCTCGATGGCGCTGACTACTC<br>TGGCACGTACGGTATCACTACCTCCGGCAACTCATTGCGCCTGAACTTCGTT

	ACCGGTTCCAACGTCGGATCTCGTACTTACCTGATGGCCGATAACACTCACT<br>ACCAAATCTTCGACTTGTTGAACCAGGAGTTCACCTTCACCGTCGATGTCTC<br>CCACCTCCCTTGCGGTTTGAACGGTGCCCTCTACTTCGTGACCATGGATGCC<br>GACGGTGGCGTCTCCAAGTACCCCAACAACAAGGCCGGTGCTCAGTACGGT<br>GTTGGATACTGTGACTCTCAATGCCCTCGTGACTTGAAGTTCATCGCTGGTC<br>AGGCCAACGTTGAGGGCTGGACGCCCTCCGCCAACAACGCCAACACTGGAA<br>TTGGCAATCACGGAGCTTGCTGCGCGGAGCTTGATATCTGGGAGGCAAACA<br>GCATCTCAGAGGCCTTGACTCCTCACCCTTGCGATACACCCGGTCTATCTGT<br>TTGCACTACTGATGCCTGCGGTGGTACCTACAGCTCTGATCGTTACGCCGGT<br>ACCTGCGACCCTGATGGATGTGACTTCAACCCTTACCGTCTTGGTGTCACTG<br>ACTTCTACGGCTCCGGCAAGACCGTTGACACCACCAAGCCCTTTACCGTTGT<br>GACTCAATTCGTCACTAACGACGGTACCTCCACCGGTTCCCTCTCCGAGATC<br>AGACGTTACTACGTTCAGAACGGCGTTGTCATCCCCCAGCCTTCCTCCAAGA<br>TCTCCGGAATCAGCGGAAATGTCATCAACTCCGACTACTGCGCTGCTGAAAT<br>CTCCACCTTTGGCGGGACTGCCTCCTTCAGCAAACACGGTGGCTTGACAAAC<br>ATGGCCGCTGGTATGGAAGCTGGTATGGTCTTGGTCATGAGTTTGTGGGACG<br>ACTACGCCGTCAACATGCTCTGGCTCGACAGCACCTACCCTACAAACGCGA<br>CTGGTACCCCCGGTGCCGCTCGTGGTACCTGCGCTACCACTTCTGGGGACCC<br>CAAGACCGTTGAATCACAATCCGGCAGCTCCTATGTCACCTTCTCTGACATT<br>CGGGTTGGTCCTTTCAATTCTACGTTCAGCGGTGGTTCTAGCACCGGTGGCA<br>GCACTACTACTACCGCCAGCCGCACCACCACCACCTCGGCCTCTTCCACCTC<br>TACTTCCAGCACCTCTACTGGCACTGGAGTCGCTGGTCACTGGGGTCAGTGT<br>GGTGGCCAGGGCTGGACTGGTCCTACCACCTGTGTTAGTGGAACCACATGC<br>ACCGTCGTGAACCCTTACTACTCTCAATGTTTGTAA
TrCBHI	ATGTATCGGAAGTTGGCCGTCATCTCGGCCTTCTTGGCCACAGCTCGTGCTC<br>AGTCGGCCTGCACTCTCCAATCGGAGACTCACCCGCCTCTGACATGGCAGA<br>AATGCTCGTCTGGTGGCACGTGCACTCAACAGACAGGCTCCGTGGTCATCG<br>ACGCCAACTGGCGCTGGACTCACGCTACGAACAGCAGCACGAACTGCTACG<br>ATGGCAACACTTGGAGCTCGACCCTATGTCCTGACAACGAGACCTGCGCGA<br>AGAACTGCTGTCTGGACGGTGCCGCCTACGCGTCCACGTACGGAGTTACCA<br>CGAGCGGTAACAGCCTCTCCATTGGCTTTGTCACCCAGTCTGCGCAGAAGA<br>ACGTTGGCGCTCGCCTTTACCTTATGGCGAGCGACACGACCTACCAGGAATT<br>CACCCTGCTTGGCAACGAGTTCTCTTTCGATGTTGATGTTTCGCAGCTGCCG<br>TAAGTGACTTACCATGAACCCCTGACGCTATCTTCTTGTTGGCTCCCAGCTG<br>ACTGGCCAATTCAAGGTGCGGCTTGAACGGAGCTCTCTACTTCGTGTCCATG<br>GACGCGGATGGTGGCGTGAGCAAGTATCCCACCAACACCGCTGGCGCCAAG<br>TACGGCACGGGGTACTGTGACAGCCAGTGTCCCCGCGATCTGAAGTTCATC<br>AATGGCCAGGCCAACGTTGAGGGCTGGGAGCCGTCATCCAACAACGCGAAC<br>ACGGGCATTGGAGGACACGGAAGCTGCTGCTCTGAGATGGATATCTGGGAG<br>GCCAACTCCATCTCCGAGGCTCTTACCCCCCACCCTTGCACGACTGTCGGCC<br>AGGAGATCTGCGAGGGTGATGGGTGCGGCGGAACTTACTCCGATAACAGAT<br>ATGGCGGCACTTGCGATCCCGATGGCTGCGACTGGAACCCATACCGCCTGG<br>GCAACACCAGCTTCTACGGCCCTGGCTCAAGCTTTACCCTCGATACCACCAA<br>GAAATTGACCGTTGTCACCCAGTTCGAGACGTCGGGTGCCATCAACCGATA<br>CTATGTCCAGAATGGCGTCACTTTCCAGCAGCCCAACGCCGAGCTTGGTAGT<br>TACTCTGGCAACGAGCTCAACGATGATTACTGCACAGCTGAGGAGGCAGAA<br>TTCGGCGGATCCTCTTTCTCAGACAAGGGCGGCCTGACTCAGTTCAAGAAG<br>GCTACCTCTGGCGGCATGGTTCTGGTCATGAGTCTGTGGGATGATGTGAGTT<br>TGATGGACAAACATGCGCGTTGACAAAGAGTCAAGCAGCTGACTGAGATGT<br>TACAGTACTACGCCAACATGCTGTGGCTGGACTCCACCTACCCGACAAACG<br>AGACCTCCTCCACACCCGGTGCCGTGCGCGGAAGCTGCTCCACCAGCTCCG<br>GTGTCCCTGCTCAGGTCGAATCTCAGTCTCCCAACGCCAAGGTCACCTTCTC<br>CAACATCAAGTTCGGACCCATTGGCAGCACCGGCAACCCTAGCGGCGGCAA

	CCCTCCCGGCGGAAACCCGCCTGGCACCACCACCACCCGCCGCCCAGCCAC TACCACTGGAAGCTCTCCCGGACCTACCCAGTCTCACTACGGCCAGTGCGGC GGTATTGGCTACAGCGGCCCCACGGTCTGCGCCAGCGGCACAACTTGCCAG GTCCTGAACCCTTACTACTCTCAGTGCCTGTAA
PoCBHII	ATGCAGAGAACATCAGCTTGGGCACTGCTCCTTCTGGCGCAGATTGCCACTG CTCAGCAGACCGTCTGGGGACAATGTGGTGGTATCGGCTACTCTGGACCGA CCAGCTGTGTTGCAGGATCTTCTTGTAGCACCCAGAACTCTTACTACGCCCA ATGTCTCCCAGGCAGTGGAAACGGCGGTGGCGGTGCGGCAACCACGACCAC GACTGCTGGACAAACCACCAAGACCACCATGGCCACCACCACCACCACTTC AACCAAGACCTCAGCTGGTAGTGGCGGCAGCACCACTACTGCTCCTCCTGCT AGCAACAGTGGCAACCCCTTCAAGGGATACCAGCCTTACGTGAACCCGTAC TACGCTTCCGAGGTTCAGAGCCTGGCTATTCCCTCTCTGGCAGCCTCTCTGG CGCCCAAGGCCAGCGCGGTGGCCAAGGTCCCATCCTTCGTTTGGCTGGACA CTGCTGCTAAGGTCCCTACTATGGGCACTTACTTGGCAGACATCAAGGCCAA GAACGCGGCTGGTGCTAACCCACCCATTGCCGGTATCTTTGTCGTTTACGAT CTTCCTGACCGTGACTGCGCTGCTCTTGCCAGTAACGGCGAGTACTCCATCG CCAACGGCGGTGTTGCCAACTACAAGAAGTACATTGACTCGATCCGCGCTC AGCTTCTCAAGTACCCTGATGTGCACACCATCCTGGTCATCGAACCCGACAG TCTCGCCAACCTGGTCACCAACATGAACGTCGCCAAATGCTCGGGTGCTCAC GACGCCTACCTGGAGTGCACTGACTATGCACTCAAGCAGCTCAACTTGCCC AACGTTGCCATGTACCTTGATGCCGGACACGCTGGCTGGCTTGGATGGCCCG CCAACATTGGACCCGCTGCCGACCTCTTCGCCAGTGTGTACAAGAATGCCG GCTCTCCCGCCGCCGTCCGTGGATTGGCCACCAACGTTGCCAACTACAACGC CTGGTCCATCTCCACCTGCCCATCTTACACTCAGGGTGACCAGAACTGTGAC GAGAAGCGCTACATCAACGCCCTCGCTCCTCTCCTCCGCGCGAACGGCTTCG ACGCCCACTTCATCATGGACACCTCCCGTAACGGTGTTCAGCCCACTAAGCA ACAAGCCTGGGGTGACTGGTGCAACGTCATTGGCACTGGCTTCGGTACCCC CTTCACCACCGACACTGGTGATGCTCTTCAGGACGCTTTCATCTGGGTCAAG CCCGGTGGTGAGTGTGACGGTACCTCGGACACATCCTCTCCTCGCTACGACG CCCACTGCGGATACAGCGATGCCCTCAAGCCGGCCCCCGAGGCTGGAACTT GGTTCCAAGCCTACTTCGAGCAGCTGCTCGTCAACGCCAACCCAAGCTTCTA A
MtCBHII	ATGGCCAAGAAGCTTTTCATCACCGCCGCGCTTGCGGCTGCCGTGTTGGCGG CCCCCGTCATTGAGGAGCGCCAGAACTGCGGCGCTGTGTGGACTCAATGCG GCGGTAACGGGTGGCAAGGTCCCACATGCTGCGCCTCGGGCTCGACCTGCG TTGCGCAGAACGAGTGGTACTCTCAGTGCCTGCCCAACAGCCAGGTGACGA GTTCCACCACTCCGTCGTCGACTTCCACCTCGCAGCGCAGCACCAGCACCTC CAGCAGCACCACCAGGAGCGGCAGCTCCTCCTCCTCCTCCACCACGCCCCC GCCCGTCTCCAGCCCCGTGACCAGCATTCCCGGCGGTGCGACCTCCACGGC GAGCTACTCTGGCAACCCCTTCTCGGGCGTCCGGCTCTTCGCCAACGACTAC TACAGGTCCGAGGTCCACAATCTCGCCATTCCTAGCATGACTGGTACTCTGG CGGCCAAGGCTTCCGCCGTCGCCGAAGTCCCTAGCTTCCAGTGGCTCGACCG GAACGTCACCATCGACACCCTGATGGTCCAGACTCTGTCCCAGGTCCGGGCT CTCAATAAGGCCGGTGCCAATCCTCCCTATGCTGCCCAACTCGTCGTCTACG ACCTCCCCGACCGTGACTGTGCCGCCGCTGCGTCCAACGGCGAGTTTTCGAT TGCAAACGGCGGCGCCGCCAACTACAGGAGCTACATCGACGCTATCCGCAA GCACATCATTGAGTACTCGGACATCCGGATCATCCTGGTTATCGAGCCCGAC TCGATGGCCAACATGGTGACCAACATGAACGTGGCCAAGTGCAGCAACGCC GCGTCGACGTACCACGAGTTGACCGTGTACGCGCTCAAGCAGCTGAACCTG CCCAACGTCGCCATGTATCTCGACGCCGGCCACGCCGGCTGGCTCGGCTGG CCCGCCAACATCCAGCCCGCCGCCGAGCTGTTTGCCGGCATCTACAATGATG CCGGCAAGCCGGCTGCCGTCCGCGGCCTGGCCACTAACGTCGCCAACTACA ACGCCTGGAGCATCGCTTCGGCCCCGTCGTACACGTCGCCTAACCCTAACTA

	CGACGAGAAGCACTACATCGAGGCCTTCAGCCCGCTCTTGAACTCGGCCGG<br>CTTCCCCGCACGCTTCATTGTCGACACTGGCCGCAACGGCAAACAACCTACC<br>GGCCAACAACAGTGGGGTGACTGGTGCAATGTCAAGGGCACCGGCTTTGGC<br>GTGCGCCCGACGGCCAACACGGGCCACGAGCTGGTCGATGCCTTTGTCTGG<br>GTCAAGCCCGGCGGCGAGTCCGACGGCACAAGCGACACCAGCGCCGCCCGC<br>TACGACTACCACTGCGGCCTGTCCGATGCCCTGCAGCCTGCCCCCGAGGCTG<br>GACAGTGGTTCCAGGCCTACTTCGAGCAGCTGCTCACCAACGCCAACCCGC<br>CCTTCTAA
TvCBHII	ATGCTGCGATATCTTTCCATTGTTGCCGCTGCGGCAATCTTGACCGGAGTGG<br>AAGCTCAGCAATCCGTTTGGGGTCAATGTGGTGGTCAAAGCTGGACTGGCG<br>CAACGTCATGCGCTGCCGGTTCTACATGCAGCATTCTTAACCCTTACTACGC<br>ACAATGTATTCCTGCCACAGCTACTTCAACCACATTGGCGACAAGCACCTCT<br>TCCACAAGTGTTGGGACGACATCGCCGCCGACAACCACCACGACGAAAGCT<br>ACTACCACAGCTACCACTGCCGCTGCATCTGGAAACCCCTTCTCTGGTTATC<br>AGCTTTATGCCAATCCGTACTACTCTTCAGAAGTACATACTCTTGCCATACC<br>ATCTCTGACTGGCACACTCGCCGCTGCTGCGACCAAGGCTGCACAGATTCCG<br>TCATTTGTCTGGCTTGACACAGCAGCTAAAGTGCCTACAATGGGTACCTACT<br>TGGCTAACATTCAAGCTGCAAACAAGGCTGGAGCTAGCCCACCTATTGCCG<br>GTATCTTCGTTGTCTATGACTTGCCTGACCGTGACTGTGCAGCTGCAGCAAG<br>TAATGGCGAATACACAGTAGCAAACAACGGTGTCGCAAACTACAAGGCTTA<br>CATCGATAGCATCGTGGCACAGTTGAAAGCTTATCCCGATGTGCACACAAT<br>CCTCATCATTGAACCCGACAGTCTAGCCAATATGGTTACCAATTTGTCTACA<br>GCCAAATGTTCCGAAGCTCAATCTGCATACTACGAGTGCGTCAACTACGCAT<br>TGATCAACCTCAACTTGGCCAACGTAGCCATGTACCTCGACGCTGGCCACGC<br>CGGTTGGCTTGGATGGTCTGCCAATCTCACACCAGCTGCTCAACTTTTTGCA<br>ACAGTCTATAAGAATGCAAGTGCTCCTGCAGCACTTCGTGGACTGGCCACC<br>AACGTTGCCAACTACAACGCTTGGTCGATCAGCAGTCCACCCTCCTACACGT<br>CTGGTGACTCCAACTACGACGAACAGCTCTATATCAATGCTTTGTCTCCTCT<br>CCTAACAGCAAACGGCTGGCCTAATGCTCACTTTATTATGGATACTTCCCGA<br>AACGGTGTTCAGCCAACCAAGCAGCAGGCATGGGGAGACTGGTGCAATGTG<br>ATCGGAACCGGCTTCGGTGTTCCTTTCACAACCAACACCGGTGACGCACTAG<br>AGGATGCCTTTGTCTGGGTCAAACCAGGTGGTGAAAGTGATGGTACCTCAA<br>ACAGTTCCTCTACTCGTTACGATTATCATTGCGGCTACAGTGATGCACTTCA<br>GCCTGCTCCCGAGGCTGGCACTTGGTTCCAAGCTTACTTTGCCCAGCTTTTG<br>ACCAATGCCAATCCAGCTTTGGCTTAG
TaEG	ATGAAGCTCGGTAGCCTCGTCCTGGCTCTGAGCGCCGCTCGTCTCACCCTGA<br>GCGCCCCCCTCGCTGACCGCAAGCAAGAGACCAAGCGCGCCAAGGTCTTCC<br>AGTGGTTCGGCAGCAACGAGTCCGGCGCCGAGTTCGGCTCCCAAAACCTGC<br>CCGGCGTGGAGGGTAAGGACTACATCTGGCCCGACCCTAACACCATCGACA<br>CCCTGATCAGCAAGGGCATGAACATCTTCCGTGTCCCCTTCATGATGGAACG<br>CCTGGTCCCCAACTCCATGACTGGTTCCCCCGACCCCAACTACCTGGCCGAC<br>CTGATCGCTACCGTCAACGCCATCACTCAAAAGGGCGCCTACGCTGTGGTTG<br>ATCCCCATAACTACGGCCGCTACTACAACTCCATCATCAGCTCCCCCTCCGA<br>CTTCCAGACCTTCTGGAAAACCGTCGCCTCCCAGTTCGCCTCCAACCCTCTC<br>GTCATCTTCGACACCAACAACGAGTACCACGACATGGACCAAACCCTGGTC<br>CTCAACCTGAACCAGGCCGCCATTGACGGCATTCGCTCCGCCGGTGCCACCT<br>CCCAGTACATCTTCGTCGAAGGTAACTCCTGGACCGGCGCCTGGACCTGGA<br>CTAACGTCAACGACAACATGAAGTCCCTCACCGATCCCAGCGACAAGATCA<br>TCTACGAGATGCACCAGTACCTCGACTCCGACGGCAGCGGCACTTCCGCTA<br>CCTGCGTCTCCTCCACCATCGGCCAGGAGCGCATCACCTCCGCCACCCAATG<br>GCTCCGCGCCAACGGCAAGAAAGGCATCATCGGCGAGTTCGCCGGTGGTGC<br>CAACGACGTCTGTGAAACCGCCATCACCGGCATGCTGGACTACATGGCTCA<br>GAACACCGACGTCTGGACCGGTGCCATTTGGTGGGCCGCCGGTCCTTGGTG

	GGGCGATTACATCTTCAGCATGGAGCCCGATAACGGCATCGCCTACCAGCA<br>GATCCTCCCCATCCTCACCCCCTATCTCTAG
TtAA9E	ATGCTCGCCAACGGCGCCATCGTCTTCCTCGCTGCTGCCCTGGGCGTCTCCG<br>GCCATTACACCTGGCCCCGTGTCAACGACGGTGCCGACTGGCAGCAAGTCC<br>GCAAGGCCGACAACTGGCAGGACAACGGTTACGTCGGCGACGTCACCTCCC<br>CCCAGATCCGCTGCTTCCAGGCTACCCCTTCCCCCGCTCCTTCCGTCCTGAA<br>CACCACCGCCGGTAGCACCGTCACCTACTGGGCCAACCCTGACGTCTACCA<br>CCCTGGCCCCGTGCAGTTCTACATGGCCCGCGTCCCTGACGGCGAGGACATC<br>AACAGCTGGAACGGCGACGGCGCCGTCTGGTTCAAGGTCTACGAGGACCAC<br>CCCACCTTCGGCGCCCAACTGACCTGGCCCAGCACCGGCAAGTCCTCCTTCG<br>CCGTCCCCATCCCTCCCTGCATCAAGTCCGGTTACTACCTGCTGCGCGCCGA<br>GCAAATTGGTCTGCACGTCGCCCAGTCCGTCGGTGGTGCCCAGTTCTACATC<br>AGCTGCGCCCAACTGTCCGTCACTGGCGGTGGCTCCACTGAGCCCCCTAACA<br>AGGTCGCCTTCCCCGGCGCTTACAGCGCTACCGACCCCGGCATCCTGATCAA<br>CATCTACTACCCCGTGCCCACCTCCTACCAGAACCCCGGTCCCGCCGTCTTC<br>TCCTGTTAG
PoBGLI	ATGAAGCTCGAGTGGCTGGAAGCCACGGTGCTTGCGGCCGCCACGGTTGCT<br>AGTGCAAAGGTATGTTGCCGAATGTACCCCCAGTTGACTTGCGATGACTCCC<br>AAGTTGTTTTTCTTTGTGTTTACTAGTGGATCTGACACAAATACTTTTGGTGA<br>TATAGGATCTTGCCTACTCTCCCCCCTTCTATCCTTCTCCATGGGCAACCGGT<br>GAAGGTGAATGGGCCGAGGCCTACAAGAAGGCTGTGGACTTTGTTTCTGGT<br>CTGACTCTTGCCGAGAAGGTCAACATCACGACCGGTGCTGGGTAGGTCCAT<br>GCGCTGAAGATGATTGCTCTGTGTATGCACATTGCGGCTGACAATTGTGTCC<br>AGATGGGAACAGGAGCGTTGTGTGGGTGAGACCGGCGGTGTCCCTCGGTAA<br>GATTGTACTCTCATCTAATATCCTCTTGGGTCCAGCAAAGGGCAAATCAAAT<br>TGACATGCGAACCGTTGAATCAGACTTGGAATGTGGGGAATGTGCATGCAA<br>GATTCTCCTCTCGGCGTTCGTAATGGTGAGACAAACTCTTTCTCAAGGATGA<br>TTCACTTCACGCGAAAGACTAACCAACCGAATGTAGCCGACTACAGCTCTG<br>CCTTCCCCGCCGGTGTGAATGTGGCTGCCACCTGGGACCGACGACTCGCGTA<br>CCAGCGTGGTACGGCCATGGGCGAGGAGCATCGCGACAAGGGTGTCGACGT<br>GCAGCTTGGCCCCGTCGCTGGTCCATTGGGCAAGAACCCCGACGGTGGTCG<br>TGGCTGGGAAGGCTTTTCTCCCGATCCGGTTCTGACCGGTGTTATGATGGCC<br>GAGACAATCAAGGGTATCCAAGATGCTGGTGTCATTGCTTGCGCCAAGCAC<br>TTCATCATGAATGAGCAGGAGCACTTCCGCCAGGCGGGTGAAGCCCAGGGA<br>TACGGATTCAATATTTCTCAGAGTTTGAGCTCCAACGTCGATGACAAGACCA<br>TGCACGAGCTGTACTTGTGGCCGTTTGTCGATTCGGTTCGGGCCGGTGTGGG<br>TTCCGTCATGTGCTCTTACAACCAGATCAACAACAGCTACGGGTGCTCCAAC<br>AGCTACACGCTCAACAAATTGCTCAAGGGCGAGCTCGGCTTTCAGGGCTTC<br>GTCATGAGCGACTGGGGTGCGCACCACAGCGGTGTCGGTGACGCCCTTGCC<br>GGTCTCGACATGTCTATGCCCGGTGATGTGATTCTTGGTAGCCCCTACTCCT<br>TCTGGGGAACTAACTTGACCGTCTCTGTGCTGAACAGCACCATCCCCGAATG<br>GCGTCTGGATGACATGGCCGTTCGTATCATGGCTGCCTACTACAAGGTCGGC<br>AGAGATCGTCATCGCACTCCTCCCAACTTCAGCTCCTGGACCCGCGATGAGT<br>ACGGCTACGAGCACTTTATTGTCCAGGAGAACTATGTCAAGCTCAACGAGC<br>GTGTCAATGTTCAACGTGATCATGCCAACGTCATCCGCAAGATTGGCTCCGA<br>CAGTATCGTGATGCTCAAGAACAACGGGGGTCTGCCTTTGACTCATCAAGA<br>GCGTCTGGTGGCTATCTTGGGCGAGGATGCTGGTTCCAACGCCTACGGCGCC<br>AACGGCTGCAGTGACCGAGGCTGTGACAACGGTACCTTGGCCATGGGCTGG<br>GGCAGTGGAACGGCCAACTTCCCCTACCTGATCACTCCCGAGCAAGCCATT<br>CAGAATGAGGTTCTCAACTACGGCAACGGTGACACCAATGTCTTTGCTGTCA<br>CAGACAACGGTGCCCTCAGCCAAATGGCTGCCCTTGCTTCAACCGCAAGTG<br>TTGCATTGGTGTTCGTCAACGCTGATTCGGGCGAGGGCTACATCAGTGTGGA<br>CGGCAACGAGGGCGATCGCAAGAACATGACCCTGTGGAAGAACGGCGAGG

	AGCTGATCAAGACCGCCACTGCCAACTGCAACAACACCATCGTCATCATGC<br>ACACCCCCAACGCCGTCCTGGTCGATTCATGGTACGACAATGAGAACATCA<br>CTGCCATTCTGTGGGCTGGTATGCCCGGCCAAGAGAGTGGTCGTAGCTTGGT<br>TGATGTTCTCTACGGCCGCACGAACCCTGGTGGCAAGACCCCCTTCACCTGG<br>GGTAAGGAGCGCAAGGATTGGGGATCTCCTCTTCTGACTAAACCCAACAAC<br>GGCCACGGTGCTCCTCAGGATGACTTCACCGATGTTCTGATTGACTATCGCC<br>GTTTCGACAAGGACAACGTGGAGCCCATCTTCGAGTTCGGCTTCGGTCTGAG<br>CTACACCAAATTTGAGTTCTCTGACATCCAGGTCAAGGCGCTGAATCACGGC<br>GAGTACAACGCCACCGTGGGCAAGACCAAGCCTGCCCCTTCGTTGGGCAAG<br>CCTGGTAATGCCTCCGATCATCTGTTCCCCAGCAACATCAACCGTGTGCGAC<br>AGTACCTTTACCCTTACCTGAACTCGACCGATCTGAAGGCGTCTGCCAACGA<br>CCCTGACTATGGCATGAATGCATCGGCGTACATTCCTCCCCATGCCACCGAC<br>AGCGACCCACAGGACCTTCTCCCCGCCAGCGGACCTTCCGGTGGCAACCCT<br>GGTTTGTTTGAGGACCTTATTGAGGTGACTGCTACTGTCACCAACACCGGCT<br>CAGTTACTGGTGACGAGGTTCCCCAGCTGGTAAGTTCTCCCGAATTCCGACT<br>CCAAGCGCTTTGCGCGAGATTGAGGTTTCTGACAGGAATGTTATATAGTACG<br>TTTCGCTTGGCGGTGCCGATGACCCCGTTAAGGTCCTCCGTGCCTTCGACCG<br>TGTCACGATCGCCCCTGGTCAGAAGCTCCGGTGGACAGCAACCCTCAACCG<br>TCGTGATCTGTCCAACTGGGATGTCCCATCACAGAACTGGATCATCTCAGAC<br>GCCCCCAAGAAGGTGTGGGTGGGCAACTCGTCGCGCAAGCTGCCTCTTTCA<br>GCCGATCTGCCCAAGGTGCAGTAA

[a]Gene sequences of TrCBHI and PoBGLI contain introns.
